# Manganese [III] Tetrakis [5,10,15,20]-Benzoic Acid Porphyrin Reduces Adiposity and Improves Insulin Action in Mice with Pre-Existing Obesity

**DOI:** 10.1371/journal.pone.0137388

**Published:** 2015-09-23

**Authors:** Jonathan R. Brestoff, Tim Brodsky, Alexandra Z. Sosinsky, Ryan McLoughlin, Elena Stansky, Leila Fussell, Aaron Sheppard, Maria DiSanto-Rose, Erin E. Kershaw, Thomas H. Reynolds

**Affiliations:** 1 Department of Health and Exercise Sciences, Skidmore College, Saratoga Springs, New York, United States of America; 2 Division of Endocrinology, Department of Medicine, University of Pittsburgh, Pittsburgh, Pennsylvania, United States of America; Monash University, AUSTRALIA

## Abstract

The superoxide dismutase mimetic manganese [III] tetrakis [5,10,15,20]-benzoic acid porphyrin (MnTBAP) is a potent antioxidant compound that has been shown to limit weight gain during short-term high fat feeding without preventing insulin resistance. However, whether MnTBAP has therapeutic potential to treat pre-existing obesity and insulin resistance remains unknown. To investigate this, mice were treated with MnTBAP or vehicle during the last five weeks of a 24-week high fat diet (HFD) regimen. MnTBAP treatment significantly decreased body weight and reduced white adipose tissue (WAT) mass in mice fed a HFD and a low fat diet (LFD). The reduction in adiposity was associated with decreased caloric intake without significantly altering energy expenditure, indicating that MnTBAP decreases adiposity in part by modulating energy balance. MnTBAP treatment also improved insulin action in HFD-fed mice, a physiologic response that was associated with increased protein kinase B (PKB) phosphorylation and expression in muscle and WAT. Since MnTBAP is a metalloporphyrin molecule, we hypothesized that its ability to promote weight loss and improve insulin sensitivity was regulated by heme oxygenase-1 (HO-1), in a similar fashion as cobalt protoporphyrins. Despite MnTBAP treatment increasing HO-1 expression, administration of the potent HO-1 inhibitor tin mesoporphyrin (SnMP) did not block the ability of MnTBAP to alter caloric intake, adiposity, or insulin action, suggesting that MnTBAP influences these metabolic processes independent of HO-1. These data demonstrate that MnTBAP can ameliorate pre-existing obesity and improve insulin action by reducing caloric intake and increasing PKB phosphorylation and expression.

## Introduction

Obesity is an increasingly prevalent metabolic disease [1] that is associated with increased risk of developing type 2 diabetes, a disease that affects approximately 24 million Americans [2] and costs an estimated $245 billion in total health care costs in the United States [3]. Oxidative stress and production of reactive oxygen species (ROS) have been linked to the development of insulin resistance and type 2 diabetes [4], suggesting a potential role for ROS in the pathogenesis of these disorders. In support of this notion, augmenting the antioxidant defense system in mice improves insulin action in the setting of obesity [5–8]. Furthermore, a large observational study of older adults demonstrated that higher total dietary antioxidant capacity was associated with improved oral glucose tolerance and insulin action [9]. Despite these associations, it remains unclear whether antioxidant supplementation or augmentation of the antioxidant defense system is therapeutically beneficial in the treatment of established metabolic disease [10,11].

The superoxide dismutases (SOD) are endogenous antioxidants that limit cellular oxidative damage and improve insulin resistance [5,6]. The cell permeable SOD mimetic, manganese (III) tetrakis [5,10,15,20] 4-benzoic acid porphyrin (MnTBAP), possesses SOD and catalase activity [12] and has been shown to reduce oxidative damage [8,13] by quenching both ROS and reactive nitrogen species [13,14]. However, studies examining the efficacy of MnTBAP on insulin resistance in rodents have produced conflicting results. Specifically, MnTBAP improves insulin action in the genetically-obese *Lep* ^*ob*^ */Lep* ^*ob*^ mouse model [8], yet MnTBAP had no effect on insulin action in wildtype mice during a short-term HFD regimen, despite protecting mice from diet-induced weight gain [15]. In addition to the lack of consensus on the effect MnTBAP on insulin action, it remains unclear whether MnTBAP reduces adiposity in mice with pre-existing obesity.

We hypothesized that MnTBAP treatment would ameliorate insulin resistance and reduce adiposity in mice with pre-existing diet-induced obesity. To test this hypothesis, we generated weight-stable diet-induced obese mice by feeding mice a high-fat diet for five months. Mice were then treated with MnTBAP or vehicle for five weeks, followed by assessment of insulin action, energy homeostasis, and adiposity. We found that MnTBAP treatment improved insulin action, in part, by increasing the phosphorylation and total expression of protein kinase B (PKB), a signaling molecule that is critical to insulin-stimulated glucose transport. We also observed markedly decreased body weight and fat mass, responses that were associated with decreased caloric intake and that likely contributed to the enhancement of insulin action. Because MnTBAP is a metalloporphyrin molecule, we hypothesized that its anti-obesity and insulin sensitizing actions may be regulated by heme oxygenase-1 (*HO-1*) [16–19], as had been shown for cobalt porphyrins [16,18–23]. Although MnTBAP treatment resulted in an up-regulation of HO-1, surprisingly pharmacologic blockade of HO-1 did not attenuate the effects of MnTBAP on body weight, adiposity, caloric intake or insulin action. Collectively, these findings reveal that MnTBAP reduces adiposity and improves insulin action in part by reducing caloric intake, a process that appears to be independent of HO-1.

## Material and Methods

### Animals

Wildtype C57BL6/J male mice (~1 month old) were purchased from the Jackson Laboratory (Bar Harbor, ME) and were randomly assigned to either a high fat diet (HFD, 58% kcal from fat) or a low fat diet (LFD, 11% kcal from fat) (Test Diets, St. Louis, MO). Mice were fed a HFD or LFD for 5 months prior to being assigned to diet- and weight-matched treatment groups. In some studies, mice then received either MnTBAP (10 mg/kg) or vehicle (2% bicarbonate) by daily intraperitoneal injection for five weeks while being maintained on their respective diets. This dose of MnTBAP has been used previously *in vivo* [8]. To test the hypothesis that MnTBAP action is dependent on HO-1, an identical study design was used, but during the 5 week intervention period HFD mice were treated with or without MnTBAP in the presence or absence of a potent HO-1 inhibitor, tin mesoporphyrin (SnMP, 20 mg/kg). This dose of SnMP has been shown to abolish HO-1 activity in tissues from mice [19]. Animal studies were conducted in accordance with the National Research Council's Guide for Care and Use of Laboratory Animals (Institute of Laboratory Animal Resources, Commission on Life Sciences, 2011). All experimental protocols were approved by the Skidmore College Institutional Animal Care and Use Committee.

### Insulin-Assisted Glucose Tolerance Test

Following an overnight fast, HFD-fed mice were subjected to an insulin-assisted glucose tolerance (IAGT) test that involves a simultaneous intraperitoneal injection of glucose (2.0 g/Kg body weight) and insulin (2.0 U/Kg body weight). The IAGT test allows for the assessment of blood glucose over a wider physiological range and avoids the severe hypoglycemia that occurs during insulin tolerance tests where only insulin is injected [24]. A handheld glucometer was used to measure blood glucose collected via the tail vein at 0, 20, 40, and 60 min following the injection.

### Caloric Intake

Caloric intake was assessed by giving mice a known amount of chow for a given period of time (3–10 days). Remaining chow was weighed and subtracted from the initial amount of chow, multiplied by the kcal/g for each diet, and expressed as kcal/day.

### Energy Expenditure

To assess energy expenditure, mice from the LFD-Vehicle, HFD-Vehicle, and HFD-MnTBAP groups were single-housed in Oxymax system metabolic cages (Columbus Instruments, Columbus, OH). After 8 h to allow for adaptation to the metabolic cages, O_2_ consumption and CO_2_ production were measured for 24 h. During this time mice had free access to water and food. All measurements were collected at room temperature. Energy expenditure during the light and dark cycles were calculated using the calorific value (3.815 +1.232*RER)*VO_2_ and expressed as kcals/day.

### Tissue Harvest

Mice were injected with insulin (2.0 U/Kg) and 10 minutes later were anesthetized with a 1:1:1 mixture of promace, ketamine hydrochloride, and xylazine by intraperitoneal injection (1.5 ml/Kg). Approximately 15 minutes following the injection of insulin, epididymal white adipose tissue (EWAT), subcutaneous white adipose tissue (SWAT), and quadriceps (QUAD) muscles were rapidly dissected, weighed, frozen in liquid nitrogen, and stored at –80°C until analysis.

### Preparation of Muscle and Adipose Tissue Extracts, Electrophoretic Analyses and Immunoblotting

Frozen QUAD muscle and EWAT were homogenized on ice in RIPA Buffer (Sigma Chemical, Inc.) (10:1 buffer to QUAD mass and 5:1 buffer to EWAT mass ratios) containing protease and phosphatase inhibitor cocktails (Halt Protease/Halt Phosphatase, Thermo Fisher). Homogenates were rotated at 4°C for 1 h and centrifuged at 9,000 x g for 30 min at 4°C. The protein concentrations of the supernatants were determined by the BCA method (Pierce, Inc), and equal amounts of protein were subjected to SDS-PAGE along with molecular weight standards (Bio-Rad, Hercules, CA and Magic Mark, Invitrogen). Proteins were then electrophoretically transferred to Immobilon membranes and immunoblotted with phospho-specific PKB antibodies (pThr^308^ PKB and pSer^473^ PKB). The pThr^308^ PKB and pSer^473^ PKB antibodies recognize PKB when phosphorylated on Thr^308^ or Ser^473^, two phosphorylation sites necessary for PKB activity [25]. Light generated by the alkaline phosphatase conjugated secondary antibody and CDP-Star reagent was detected using a digital imaging system (UVP, Upland, CA). Phospho-specific immunoblots were stripped and re-probed with PKB-α or PKB-ß antibodies. To account for gel loading differences, all immunoblots were stripped and re-probed with a GAPDH or α-tubulin antibody. Relative signal intensities of immunoreactive bands were determined using Total Lab software (Nonlinear, Inc., Durham, NC). All data were normalized to the appropriate loading control and expressed as a percentage of LFD-Vehicle. The GAPDH antibody was from Abcam (Cambridge, MA), the phospho-specific PKB and α-tubulin antibodies were from Cell Signaling Technology (Beverly, MA), and the PKB-α and PKB-ß antibodies were generous gifts from Dr. Morris J. Birnbaum (University of Pennsylvania).

### RNA Extraction and Real Time Quantitative PCR

Total RNA was extracted from EWAT using an RNA extraction kit for lipid-rich tissues (Qiagen). RNA concentrations were measured using a spectrophotometer (NanoDrop, Thermo Scientific). A 1 ug aliquot of total RNA was reverse transcribed using the RETROscript kit from Ambion (Austin, TX). Quantitative polymerase chain reaction (qPCR) was performed using cDNA and TaqMan primers for *Ho-1* (Mm00516005_m1) and *Gapdh* (Mm99999915_g1) using a StepOne Plus Real-Time PCR System (Applied Biosystems, Foster City, CA). Relative quantitation of amplified cDNA targets were determined by the delta-delta cycle threshold (ΔΔCT) method.

### Statistical Analysis

A 2 x 2 (diet x treatment) analysis of variance (ANOVA) with repeated measures was used to detect statistically significant effects of MnTBAP on body weight, caloric intake, and blood glucose levels during the IAGT. A two-way ANOVA was used to detect statistically significant effects of MnTBAP on EWAT mass, gene expression and area under the IAGT curve. A one-way ANOVA was used to detect statistically significant effects of MnTBAP on energy expenditure, oxygen consumption, and RER. A one-way ANOVA with repeated measures was used to detect statistically significant effects of HO-1 inhibition on body weight, caloric intake, and IAGT, and a one-way ANOVA was used to detect statistically significant effects of HO-1 inhibition on EWAT mass and area under the IAGT curve. Following a significant F ratio and inspection of interactions, *a priori* mean comparisons were conducted using Fisher’s least significant difference (LSD) post-hoc test. Data are expressed as means ± SEM or % LFD-Vehicle ± SEM, and the level of statistical significance was set at p < 0.05.

## Results

### MnTBAP Treatment Decreases Pre-Existing Obesity by Altering Energy Homeostasis

**Fig 1:**
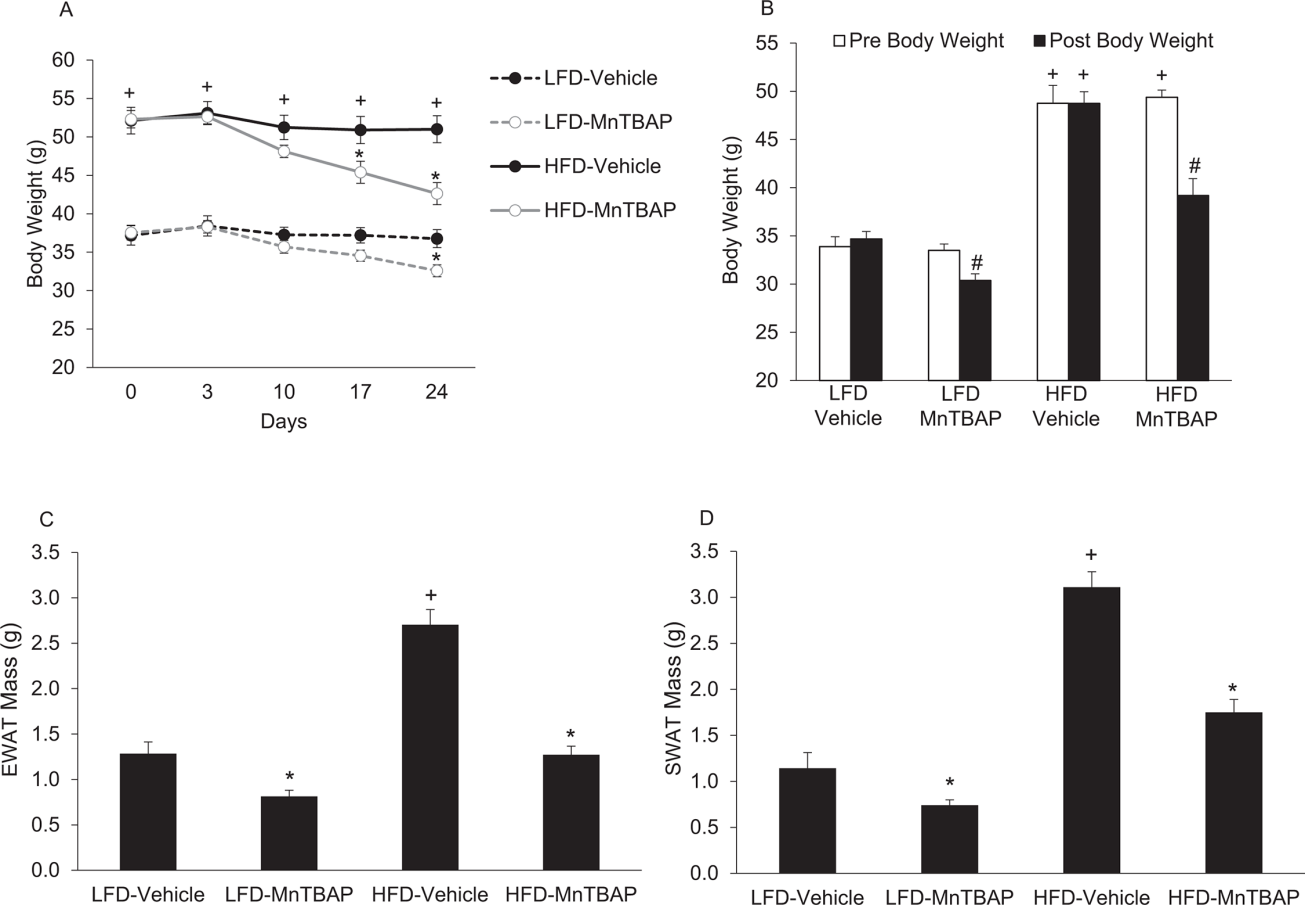
MnTBAP treatment reduces body weight and adipose tissue mass in mice fed a LFD or HFD. Mice were fed a LFD or HFD for 5 months and then treated with or without MnTBAP (10 mg/kg) daily for 5 weeks. **(A)** Fed-state body weights at different treatment durations. **(B)** Fasted body weights before and after MnTBAP or vehicle treatments. **(C)** Epididymal white adipose tissue (EWAT) mass. *, Denotes statistically significant difference from respective vehicle-treated mice. ^+^, Denotes statistically significant difference from respective LFD mice. N = 5–6 mice per group.

We first determined the effect of MnTBAP treatment on overall energy homeostasis in mice previously fed a LFD or HFD for 6 months. As expected, body weight was higher in HFD mice compared to LFD mice prior to initiating MnTBAP treatment (Fig 1A and 1B). In both HFD and LFD mice, treatment with MnTBAP resulted in significant weight loss compared to vehicle-treated counterparts (Fig 1A and 1B). MnTBAP treatment significantly decreased EWAT and SWAT (Fig 1C and 1D) mass in mice fed either a LFD or HFD. Remarkably, MnTBAP treatment reduced EWAT and SWAT mass in HFD-fed mice to levels comparable to LFD-Vehicle mice (Fig 1C and 1D). Taken together, our data indicate that MnTBAP treatment reduces body weight and adipose tissue mass in mice with pre-existing obesity.

**Fig 2:**
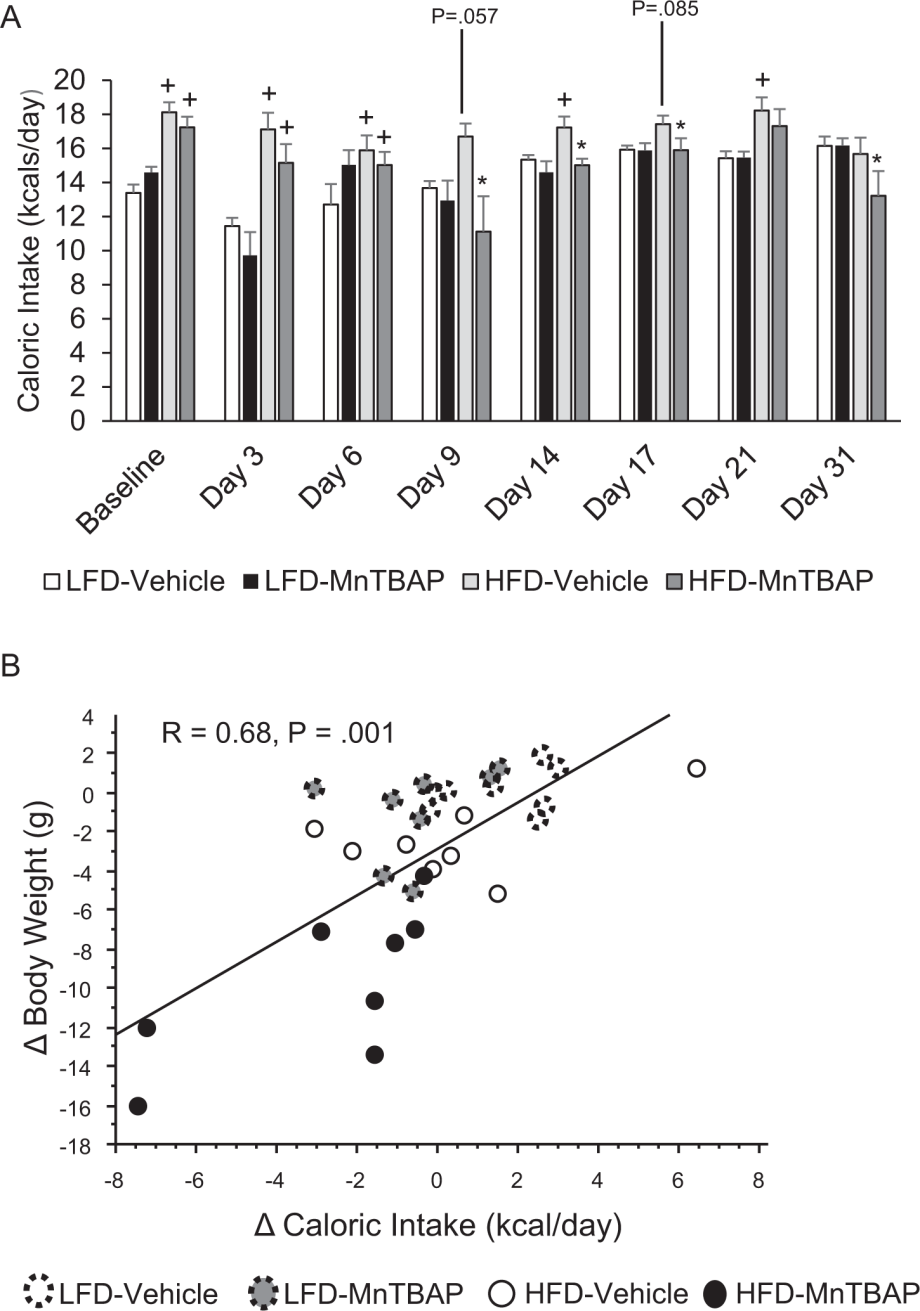
MnTBAP treatment reduces caloric intake in mice fed a HFD. **(A)** Caloric intake was assessed at various time points of MnTBAP treatment in mice previously fed a LFD or HFD for 5 months. **(B)** Correlation between the change in body weight and the change in caloric intake from pre-treatment to post-treatment. *, Denotes statistically significant difference from HFD-Vehicle. ^+^, Denotes statistically significant difference from respective LFD mice. P-value for post-hoc analysis for LFD-Vehicle vs. HFD-Vehicle mice: Day 9, P = 0.057; Day 17, P = 0.085. N = 6–8 mice per group.

**Fig 3:**
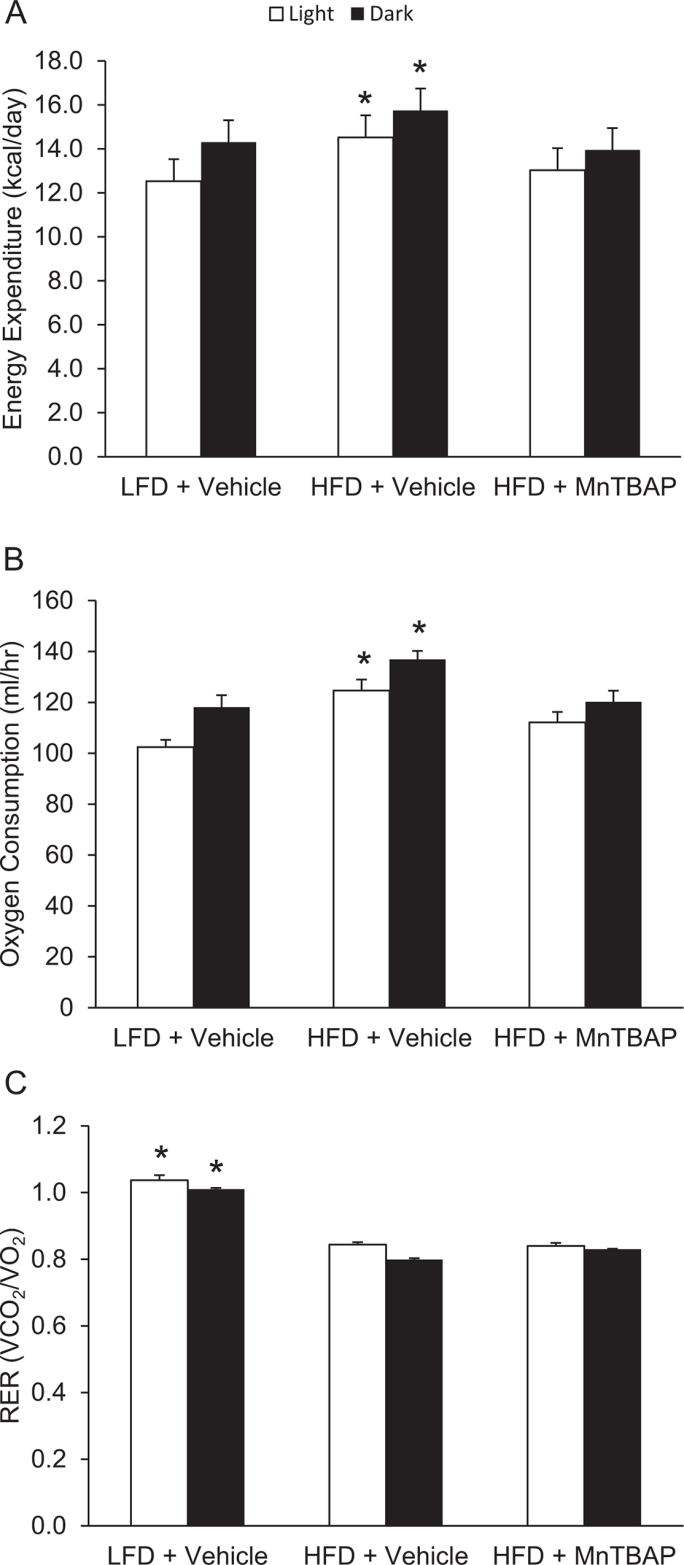
The effects of a HFD and MnTBAP treatment on energy expenditure, oxygen consumption, and RER in mice. Indirect calorimetry using open circuit spirometry was conducted for 24 hours 3 weeks into the MnTBAP treatment course for the assessment of energy expenditure **(A),** oxygen consumption **(B),** and RER values **(C)**. *, Denotes statistically significant difference from the all other groups respective of light/dark cycle. N = 6 mice per group.

Next, we determined if the anti-obesity action of MnTBAP was due to changes in caloric intake and/or energy expenditure. Although MnTBAP decreased body weight and fat mass in both HFD and LFD mice, MnTBAP treatment was associated with a significant ~10% decrease in caloric intake in HFD mice only (Fig 2A). In addition, there was a strong correlation between the changes in body weight and the changes in caloric intake (Fig 2B). Unlike caloric intake, changes in energy expenditure do not appear to be increased in mice treated with MnTBAP or account for the reduction in adiposity (Fig 3A and 3B). Furthermore, there were no differences in RER (Fig 3C) between HFD-Vehicle and HFD-MnTBAP. Collectively, these data suggest that decreased caloric intake contributes to MnTBAP-induced weight loss in mice with pre-existing obesity.

### MnTBAP Treatment Improves Insulin Action

**Fig 4:**
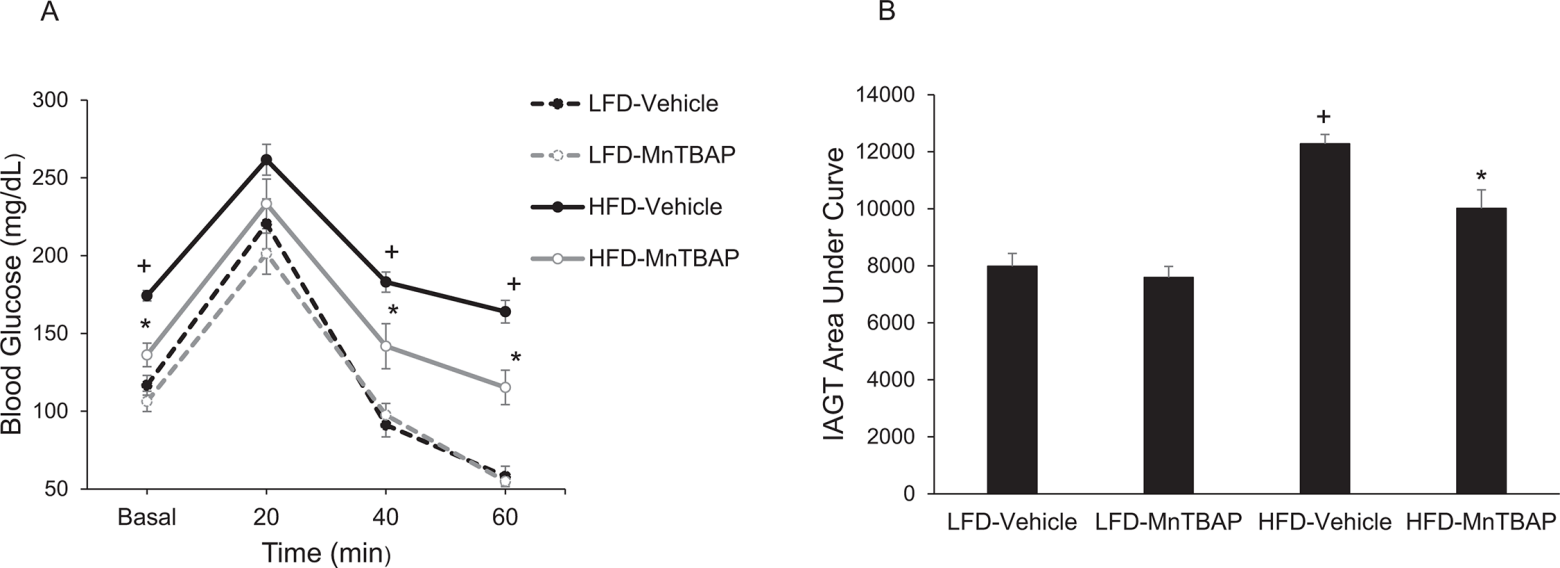
MnTBAP treatment improves insulin-assisted glucose tolerance (IAGT) in mice fed a HFD. Mice were simultaneously injected with 2.0 U/Kg insulin and 2.0 g/Kg glucose and blood glucose values were assessed at baseline and 20, 40, and 60 min following the injection (Panels A) and the area under the IAGT curve was calculated (Panels B). *, Denotes statistically significant difference from HFD-Vehicle. ^+^, Denotes statistically significant difference from LFD-Vehicle mice. N = 7–14 mice per group.

*In vivo* insulin action was determined by conducting IAGT tests that detects diet-induced insulin resistance at more physiologically-relevant blood glucose levels than insulin tolerance tests [24]. Blood glucose values were higher at baseline and during the IAGT test in mice fed a HFD compared to a LFD, indicating the presence of diet-induced insulin resistance (Fig 4A and 4B). In HFD mice treated with MnTBAP, blood glucose levels were lower at baseline and during the IAGT test compared to HFD mice treated with vehicle (Fig 4A and 4B), suggesting that MnTBAP improves insulin resistance in mice with pre-existing diet-induced obesity. MnTBAP did not alter blood glucose levels during the IAGT test in LFD mice (Fig 4A and 4B).

**Fig 5:**
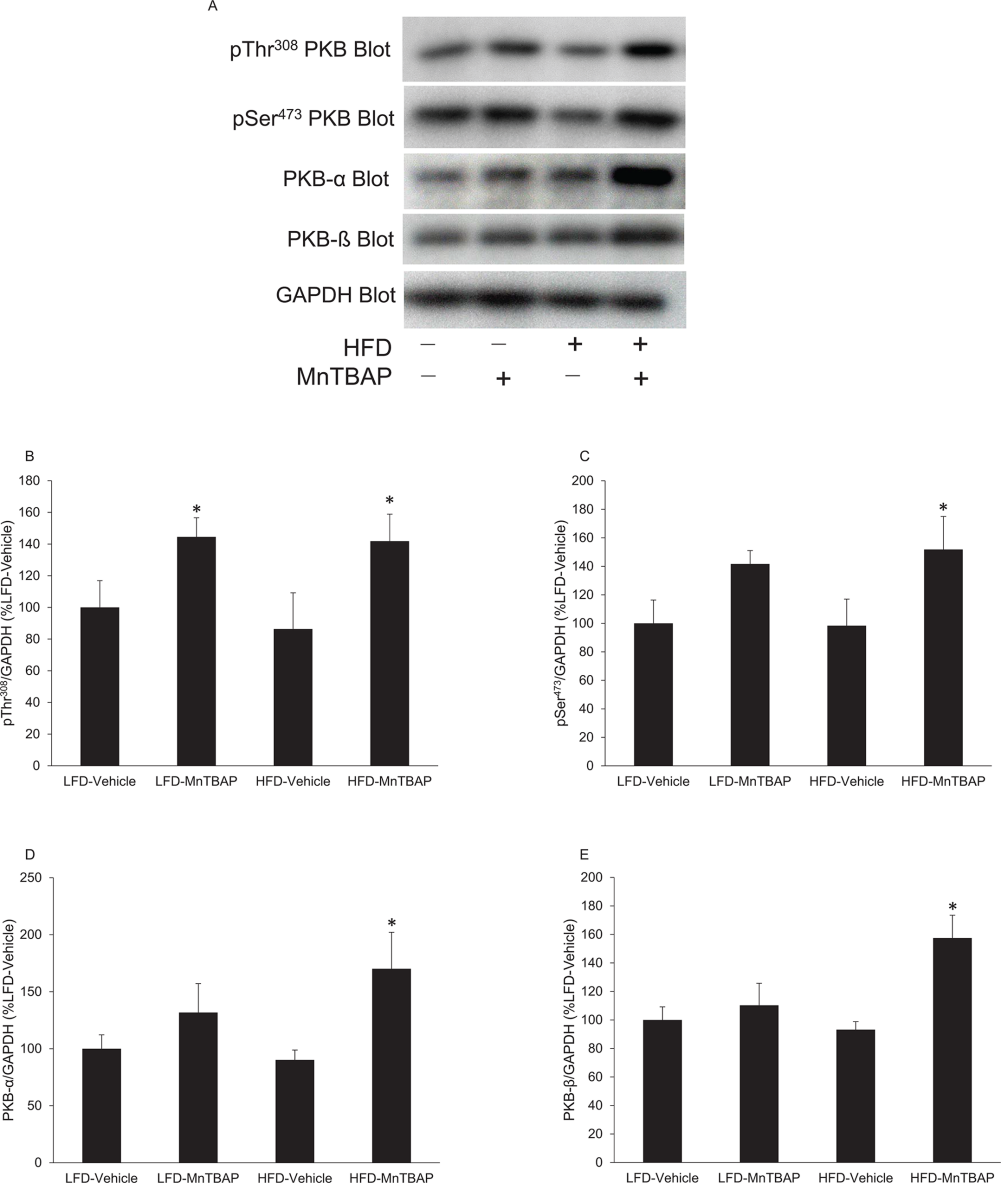
MnTBAP treatment increase insulin-stimulated PKB phosphorylation and content in muscles from mice fed a HFD. Mice were fed a LFD or HFD for 5 months and then treated with or without MnTBAP (10 mg/kg) daily for 5 weeks. At 15 min following an intraperitoneal injection of insulin (2 U/Kg), quadriceps muscles were excised and rapidly frozen in liquid nitrogen for subsequent Western blot analyses. **(A)** Representative PKB immunoblots for pThr^308^ and pSer^473^ PKB as well as total PKB-α and PKB-β isoforms. GAPDH was used as a loading control. Quantification of **(B)** pThr^308^ PKB, **(C)** pSer^308^ PKB, **(D)** PKB-α and **(E)** PKB-β content, each normalized to GAPDH. *, Denotes statistically significant difference from HFD-Vehicle. N = 5–6 mice per group.

To test whether this effect was associated with enhanced insulin action in skeletal muscle and white adipose tissue, we evaluated PKB, a key molecule in the insulin signaling cascade that is activated by phosphorylation at Thr^308^ and Ser^473^. Following insulin stimulation, both pThr^308^ and pSer^473^ immunoreactivity were significantly greater in muscles from HFD mice treated with MnTBAP compared to vehicle controls (Fig 5A–5C). To determine whether the observed increase in PKB phosphorylation was due to phosphorylation *per se* versus increased content of PKB, we also determined PKB-α and β levels. PKB-α (Fig 5D) and PKB-β (Fig 5E) immunoreactivities were higher in muscles from HFD mice treated with MnTBAP compared to muscles from HFD mice treated with vehicle. These results indicate that MnTBAP enhances insulin signaling in muscle by increasing the total content of PKB.

**Fig 6:**
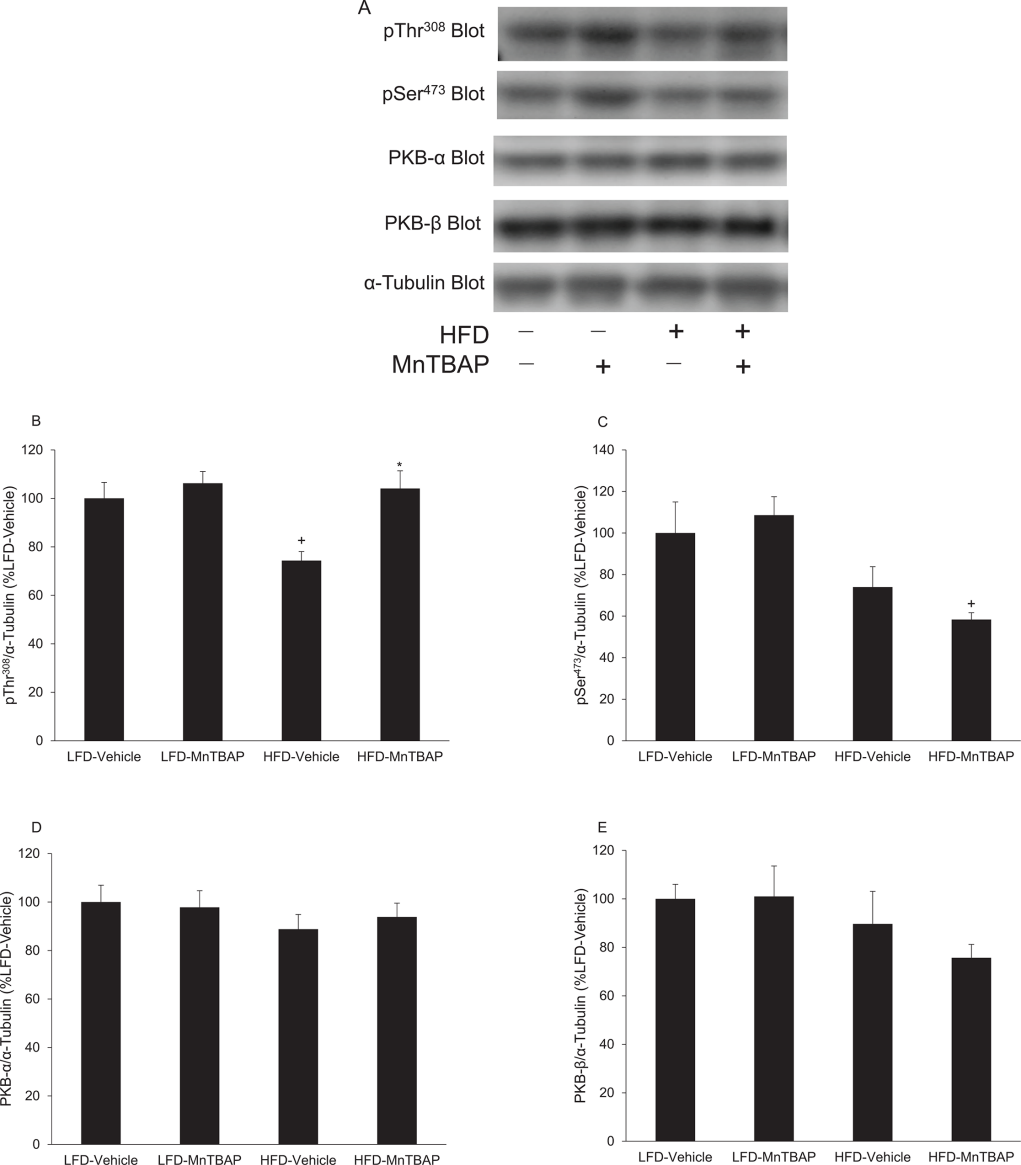
MnTBAP treatment alters insulin-stimulated PKB phosphorylation but not content in EWAT from mice fed a HFD. Mice were fed a LFD or HFD for 5 months and then treated with or without MnTBAP (10 mg/kg) daily for 5 weeks. At 15 min following an intraperitoneal injection of insulin (2 U/Kg), EWAT was excised and rapidly frozen in liquid nitrogen for subsequent Western blot experiments. **(A)** Representative PKB immunoblots for pThr^308^ and pSer^473^ PKB as well as total PKB-α and PKB-β isoforms. α-tubulin was used as a loading control. Quantification of **(B)** pThr^308^ PKB, **(C)** pSer^308^ PKB, **(D)** PKB-α and **(E)** PKB-β content, each normalized to α-tubulin. *, Denotes statistically significant difference from HFD-Vehicle. ^+^, Denotes statistically significant difference from LFD-MnTBAP. N = 5–6 mice per group.

The effects of MnTBAP on PKB phosphorylation and content differed in EWAT compared to muscle. Specifically, in EWAT of HFD mice, MnTBAP treatment increased the phosphorylation of Thr^308^ on PKB compared to vehicle-treated controls (Fig 6B). This effect was not observed for the Ser^473^ site on PKB (Fig 6C). Further, the content of PKB-α and β isoforms in EWAT was not altered by MnTBAP treatment (Fig 6D and 6E). These results in EWAT suggest that MnTBAP treatment improves diet-induced insulin resistance, in part, by increasing insulin-stimulated phosphorylation of PKB on Thr^308^. Further, these data suggest that the effects of MnTBAP on insulin action are tissue-specific.

### MnTBAP Treatment Increases Adipose Tissue Ho-1 Gene Expression

**Fig 7:**
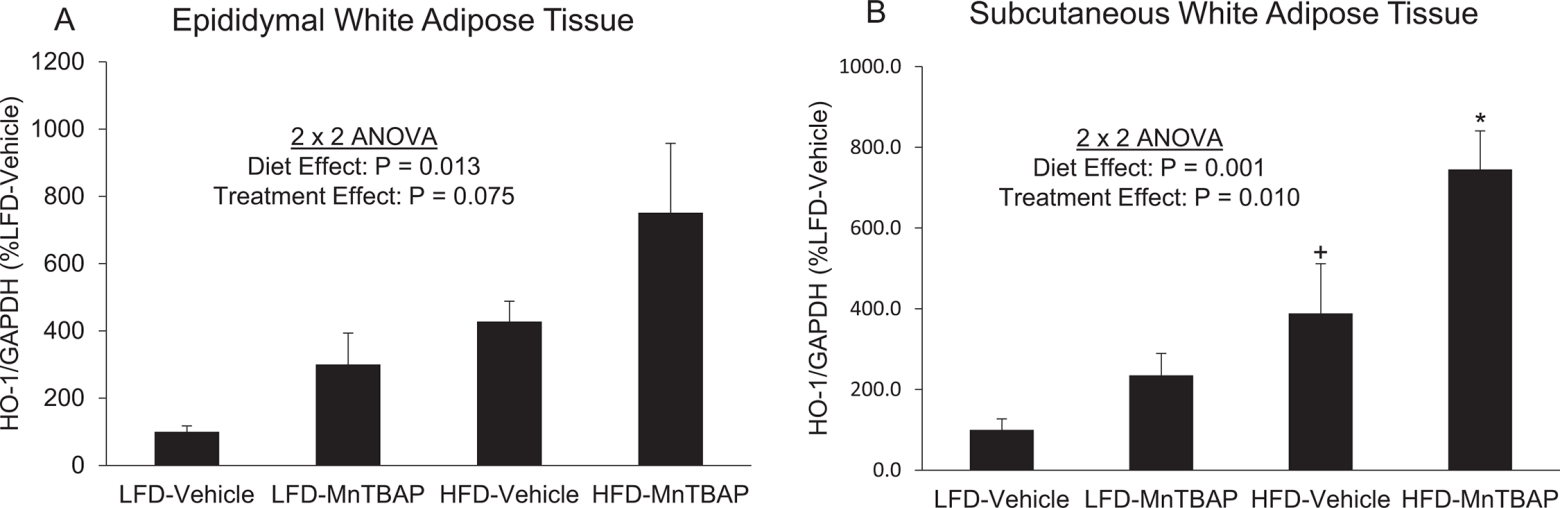
MnTBAP treatment increases *Ho-1* mRNA levels in adipose tissue from mice fed a HFD. The mRNA levels for Ho-1 from EWAT (A) and SWAT (B) of mice fed a LFD or HFD and treated with MnTBAP or vehicle. ^+^, Denotes statistically significant difference from LFD-Vehicle. *, Denotes statistically significant difference from HFD-Vehicle.

HO-1 is an enzyme induced by metalloporphyrin molecules that has been shown to reduce adiposity and insulin resistance [16–19]. Further, previous studies have shown that cobalt metalloporphyrins can decrease obesity in an HO-1-dependent manner [16,18–23]. Therefore, we hypothesized that the anti-obesity and insulin-sensitizing properties of MnTBAP are mediated by HO-1. To investigate this, we first tested whether MnTBAP treatment increased HO-1 mRNA levels in EWAT and SWAT from mice fed a HFD or LFD. As expected, mice that received MnTBAP had elevated *Ho-1* levels in EWAT (Fig 7A: MnTBAP main effect, P = 0.075) and SWAT (Fig 7B, MnTBAP main effect, P = 0.01, post-hoc P = 0.01) compared to adipose tissue from mice that received vehicle. These results suggest that MnTBAP increases *HO-1* expression in adipose.

### SnMP Does Not Block MnTBAP-Induced Changes in Adiposity, Caloric Intake, and Insulin Action

**Fig 8:**
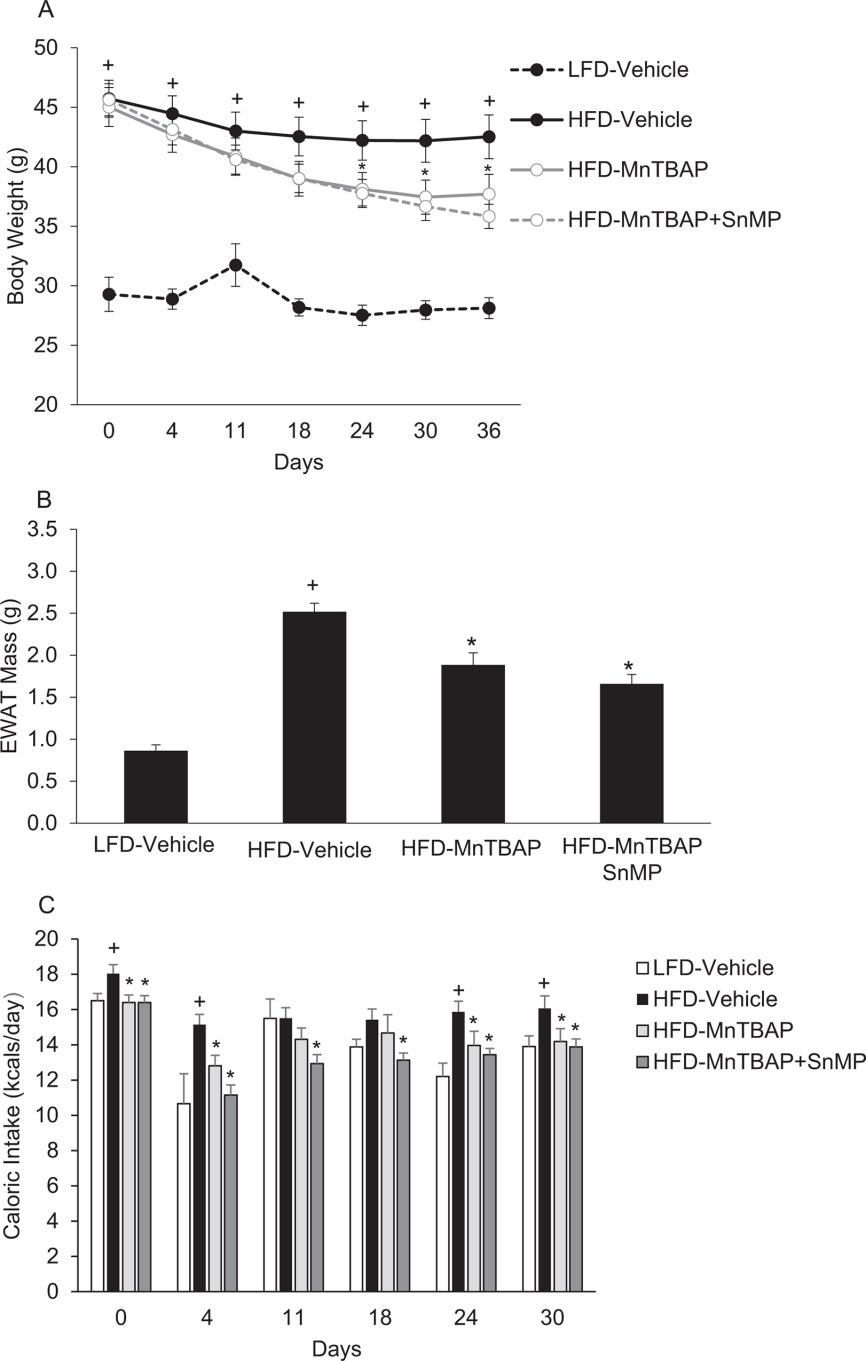
The HO-1 inhibitor SnMP does not block MnTBAP’s ability to reduce body weight, adipose tissue mass, and caloric intake in mice fed HFD. Mice were fed a LFD or HFD for 5 months and then treated with MnTBAP (10 mg/kg) alone or in combination with SnMP (20 mg/Kg) daily for 5 weeks. **(A)** Fed-state body weights at different treatment durations. **(B)** Epididymal white adipose tissue (EWAT) mass. (C) Caloric intake was assessed at various time points of MnTBAP or MnTBAP+SnMP treatment in mice previously fed a LFD or HFD for 5 months. *, Denotes statistically significant difference from LFD vehicle-treated mice. ^+^, Denotes statistically significant difference from respective LFD mice. N = 5–6 mice per group.

**Fig 9:**
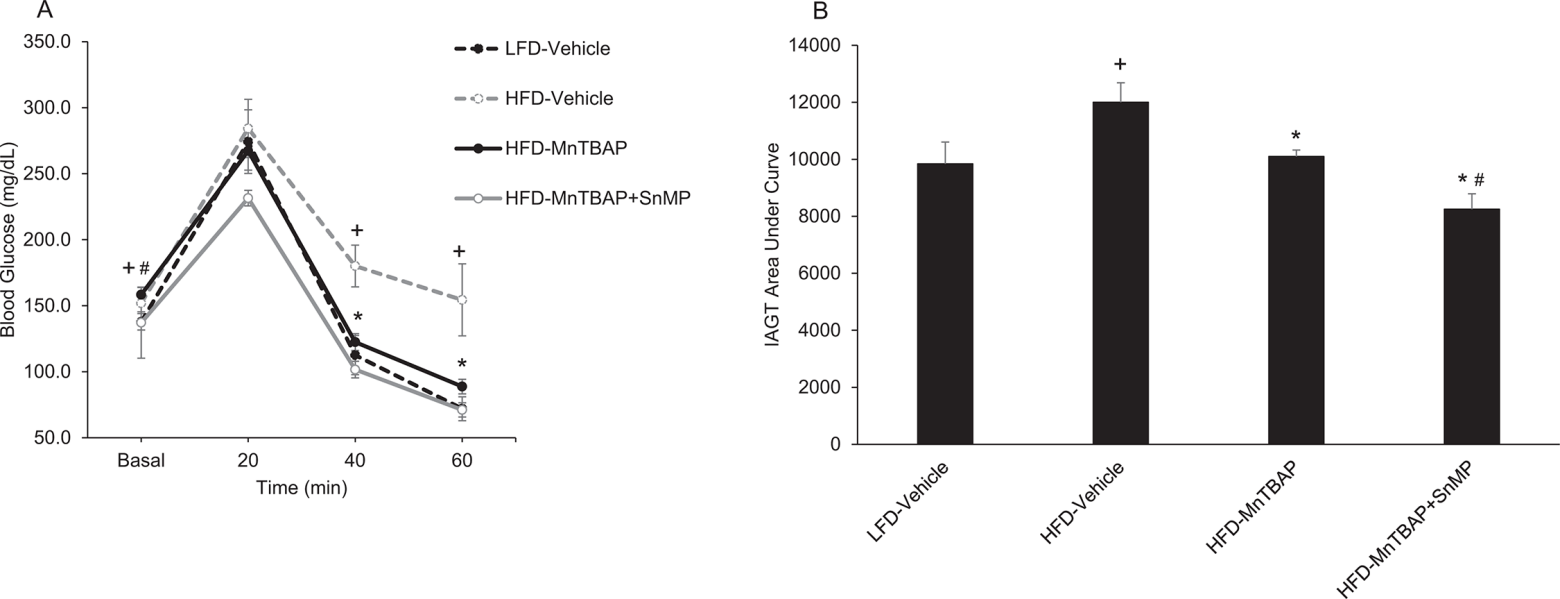
SnMP improves MnTBAP’s ability to reduce blood glucose levels during IAGT tests. Mice were fed a LFD or HFD for 5 months and then treated with MnTBAP (10 mg/kg) alone or in combination with SnMP (20 mg/Kg) daily for 5 weeks. Mice were simultaneously injected with 2.0 U/Kg insulin and 2.0 g/Kg glucose and blood glucose values were assessed at baseline and 20, 40, and 60 min following the injection (Panels A) and the area under the IAGT curve was calculated (Panels B). *, Denotes statistically significant difference from HFD-Vehicle. ^+^, Denotes statistically significant difference from LFD-Vehicle mice. ^#^, Denotes statistically significant difference from HFD-MnTBAP mice. N = 5 mice per group.

To further address the hypothesis that HO-1 mediates the anti-obesity and insulin sensitizing actions of MnTBAP, we next tested whether blockade of HO-1 activity with tin mesoporphyrin (SnMP) (17), a potent HO-1 inhibitor, would prevent the ability of MnTBAP to limit obesity and improve insulin resistance. HFD-fed mice that received both MnTBAP and SnMP experienced similar decreases in bodyweight, EWAT mass, and caloric intake compared to HFD-fed mice that received only MnTBAP (Fig 8A–8C). HFD-fed mice treated with both MnTBAP and SnMP exhibited further enhancement of insulin sensitivity compared to HFD-fed mice that received only MnTBAP (Fig 9A and 9B). These data suggest that MnTBAP limits obesity or ameliorates insulin resistance in an HO-1 independent manner.

## Discussion

To date, there is conflicting evidence regarding the effect of MnTBAP on adiposity and *in vivo* insulin action [8,15], and it remains unknown whether MnTBAP can ameliorate pre-existing obesity and insulin resistance by altering energy homeostasis. Further, the mechanisms by which MnTBAP improves metabolic dysfunction are poorly understood. The present study demonstrates that MnTBAP treatment markedly decreases pre-existing obesity and insulin resistance by decreasing caloric intake. MnTBAP also improves insulin action by increasing the phosphorylation and total content of PKB, a signaling molecule that is critical for insulin-stimulated glucose transport [26]. Although MnTBAP treatment increased *HO-1* expression in adipose tissue, this response does not appear to mediate the effects of MnTBAP on body weight, adiposity, caloric intake or insulin action.

Pires et al. [15] recently demonstrated that MnTBAP lowered ROS in adipose tissue and prevented weight gain during short-term HFD feeding without changes in energy expenditure or caloric intake. The present study expands the findings of Pires et al. [15] by showing that MnTBAP reduces adiposity in mice with pre-existing diet-induced obesity. Unlike Pires et al. [15] we observed that MnTBAP significantly decreases caloric intake and provide evidence suggesting that decreased caloric intake may explain approximately 46% of MnTBAP-induced weight loss (R = 0.68, R^2^ = 0.46, Fig 3). These data led us to conclude that other mechanisms also likely contribute to MnTBAP-induced weight loss. Although MnTBAP treatment was not associated with increased energy expenditure, we found that MnTBAP increased expression of the antioxidant-responsive gene *HO-1* in adipose tissue (Fig 7), which has been previously been shown to limit obesity and insulin resistance [16,19,21]. Previous studies have shown that some metalloporphyrins can induce HO-1 expression and promote weight loss and that pharmacologic treatment with the potent HO-1 inhibitor SnMP blocks this weight loss effect [16,18–23]. In one study the beneficial metabolic effects of metalloporphyrin-induced HO-1, at least in the liver, were shown to be mediated by sirtuin-1 (SIRT1), a deacetylase that has important roles in metabolism [27,28]. We therefore hypothesized that the beneficial metabolic effects of treating mice with MnTBAP, a metalloporphyrin, are mediated by HO-1. However, surprisingly, we observed that blockade of HO-1 activity with SnMP did not affect the ability of MnTBAP to decrease body weight, adiposity or caloric intake or to enhance insulin action. Future studies will be required to decipher the mechanism responsible for MnTBAP-induced reductions in caloric intake.

The ability of MnTBAP to enhance insulin action in mice with pre-existing diet-induced obesity in our studies is consistent with prior work indicating that MnTBAP improves insulin action in genetically obese ob/ob mice [8]. In contrast, Pires et al. observed no changes in insulin action following MnTBAP treatment in mice fed a HFD for 5 weeks [15], a discrepancy that may be related to the different mechanisms of insulin resistance that occur in acute versus chronic HFD feeding [29]. In our studies, the MnTBAP-induced improvements in insulin action is likely partially explained by the substantial reduction in adiposity observed following MnTBAP treatment. It is well-established that weight loss produced by decreasing caloric intake improves insulin action, and we demonstrate that MnTBAP treatment reduced caloric intake and improved insulin action in mice fed a HFD but not in mice fed a LFD. Although the reduction in caloric intake likely contributes to increased insulin action in mice treated with MnTBAP, we show that MnTBAP treatment significantly increases PKB-α and PKB-β content in skeletal muscle, resulting in greater insulin-stimulated phosphorylation of PKB. This effect may be independent of caloric intake because previous studies have demonstrated that caloric restriction does not affect total PKB content [30,31]. In addition to the increase in PKB content in skeletal muscle, we demonstrated an increase in PKB phosphorylation on Thr^308^ in EWAT without changes in the total abundance of the protein in HFD mice treated with MnTBAP. These results in EWAT are likely a consequence of reduced caloric intake and the subsequent loss of adiposity [30,31]. Taken together, these data suggest that MnTBAP has tissue specific effects on PKB signaling pathways in skeletal muscle and white adipose tissue. The mechanism by which MnTBAP increases the total content of PKB specifically in skeletal muscle is unclear and warrants further study.

## Conclusions

In summary, we have shown that treating mice with MnTBAP ameliorates diet-induced obesity and improves *in vivo* insulin action. Almost 50% of the weight loss effect of MnTBAP is due to decreased caloric intake and, unlike other metalloporphyrins, appears to be independent of HO-1. Further, the decrease in adiposity following MnTBAP treatment appears to contribute to enhanced insulin sensitivity, an effect that may mediated by tissue-specific changes in PKB content and phosphorylation. Overall, these data demonstrate that MnTBAP promotes weight loss and enhances insulin action by reducing caloric intake and increasing PKB activity. In conclusion, our results indicate that MnTBAP and possibly other SOD mimetics not only offer a new treatment strategy for obesity and insulin resistance, but may also prove effective against muscle wasting conditions where PKB activity is impaired.

Obesity and insulin resistance have both been associated with the over-production of ROS and oxidative stress [4]. This association led to investigations that demonstrated improved insulin action by enhancing the antioxidant defense system [5–8]. Although antioxidants can reduce oxidative stress and improve insulin action [5,6,8], this effect was thought to be independent of changes in adiposity until Pires et al. [15] recently demonstrated that MnTBAP reduced ROS and prevented weight gain during a short-term HFD regimen in lean mice. The present study expands the work of Pires et al [15] by showing MnTBAP reverses pre-existing obesity following long-term HFD feeding. Taken together, Pires et al and the present study establish MnTBAP and possibly other SOD mimetics as a new avenue for the treatment of obesity.
